# A field guide to whole-genome sequencing, assembly and annotation

**DOI:** 10.1111/eva.12178

**Published:** 2014-06-24

**Authors:** Robert Ekblom, Jochen B W Wolf

**Affiliations:** Department of Evolutionary Biology, Uppsala UniversityUppsala, Sweden

**Keywords:** bioinformatics, conservation genomics, genome assembly, next generation sequencing, vertebrates, whole - genome sequencing.

## Abstract

Genome sequencing projects were long confined to biomedical model organisms and required the concerted effort of large consortia. Rapid progress in high-throughput sequencing technology and the simultaneous development of bioinformatic tools have democratized the field. It is now within reach for individual research groups in the eco-evolutionary and conservation community to generate *de novo* draft genome sequences for any organism of choice. Because of the cost and considerable effort involved in such an endeavour, the important first step is to thoroughly consider whether a genome sequence is necessary for addressing the biological question at hand. Once this decision is taken, a genome project requires careful planning with respect to the organism involved and the intended quality of the genome draft. Here, we briefly review the state of the art within this field and provide a step-by-step introduction to the workflow involved in genome sequencing, assembly and annotation with particular reference to large and complex genomes. This tutorial is targeted at scientists with a background in conservation genetics, but more generally, provides useful practical guidance for researchers engaging in whole-genome sequencing projects.

## Introduction

The field of conservation genetics is concerned with studying genetic and evolutionary processes in the context of biodiversity conservation (Frankham et al. 2010). Traditionally, a small number of neutral genetic markers were employed to study patterns of genetic variation of individuals and populations with the aim to explore underlying processes and their relevance to conservation. Marker-based measures provide insight into effective population sizes (*N*_*e*_), recent demographic events (e.g. bottlenecks and expansions), genetic relatedness and the level of inbreeding. Genetic markers are also routinely employed in monitoring schemes (e.g. identification of individuals and capture–recapture modelling) and in breeding programs (Romanov et al. 2009) and have been extensively used to characterize population substructuring and genetic connectivity, to delineate conservation units and to infer interspecific admixture events (Höglund 2009; Allendorf et al. 2013). Under the premise of a causal relationship between neutral genetic variation and population viability, these data can inform practical conservation decisions (Frankham 1995).

**Figure 1:**
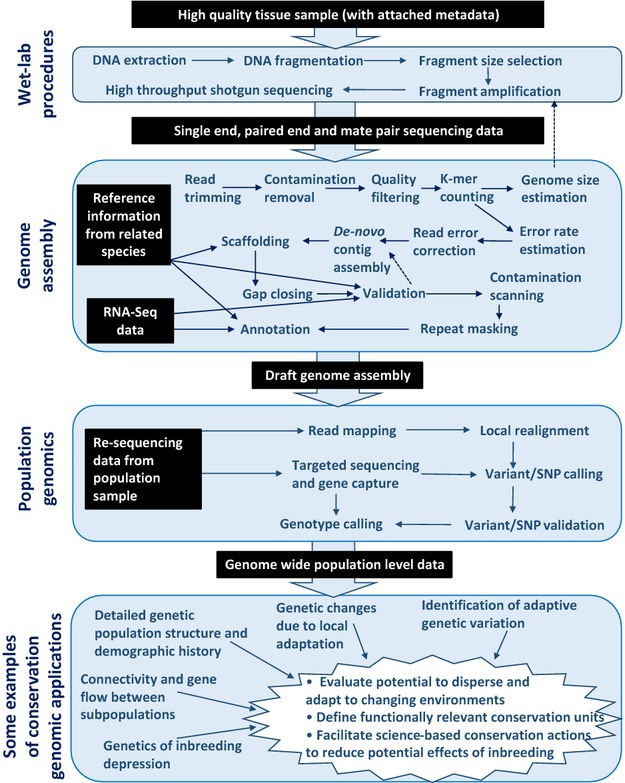
Workflow of a typical *de novo* whole-genome sequencing project. Black boxes with white text indicate genomic resources becoming available during the course of the project. From the top: wet-lab procedures, *de novo* assembly bioinformatic pipeline, postassembly analyses of additional population-wide sampling (population genomics), conservation genomic questions to address and analyses to perform (conservation genomic applications). Bullet points within the white star in the bottom part of the figures represent ultimate goals in conservation biology that can be addressed using genomic information combined with high-quality ecological data.

Rapid advances in sequencing technology and bioinformatic tools during the last decade have initiated a transition from classical conservation genetics to conservation genomics (Fig.1; Primmer 2009; Allendorf et al. 2010; Ouborg et al. 2010; Steiner et al. 2013). This development has two major implications. First, by significantly scaling-up the number of genetic markers, genomewide approaches enhance the power and resolution for the above-mentioned applications and improve the reliability of conclusions (Steiner et al. 2013). Second, the application of genomic technologies opens novel axes of investigation (Allendorf et al. 2010; Ouborg et al. 2010). Genome-scale data provide information beyond neutral genetic variation or candidate gene approaches (e.g. major histocompatibility complex genes; Hedrick 1999) and thus enable screening for selectively important variation and assessing the adaptive potential of populations (Primmer 2009). For example, approaches such as genomewide scans for selection, association mapping or quantitative trait loci (QTL) mapping can pinpoint loci of relevance for local adaptation of the target population (Steiner et al. 2013), with the potential to conserve evolutionary processes – a long sought after goal in conservation biology (Crandall et al. 2000; Fraser and Bernatchez 2001). Genomewide analyses further allow addressing the poorly understood mechanistic basis of inbreeding depression (epistasis, directional dominance versus overdominance, many versus few loci), or assessing the impact of genetic variation on patterns of gene expression, and plastic response to environmental change. Genomic approaches can also be applied to highly fragmented DNA from ancient material (e.g. from museum specimens; Pääbo et al. 2004; Bi et al. 2013), to characterize environmental samples (Shokralla et al. 2012) and to understand how environmental perturbations affect microbial communities (Mardis 2008), representing largely unexplored terrain in conservation biology.

The above-mentioned applications do not necessarily require a reference genome sequence. Many analyses, including taxonomic delineation, characterization of demographic events, estimates of inbreeding or relatedness, can be successfully conducted in the absence of a genome reference. Instead, large-scale maker data such as genotyping-by-sequencing (Elshire et al. 2011), RAD-Seq (Narum et al. 2013), reduced representation sequencing (Van Tassell et al. 2008), amplicon sequencing (Zavodna et al. 2013) or transcriptome sequencing (Ekblom and Galindo 2011) can be effectively utilized without relying on a genome backbone. A complete and well-annotated genome sequence, however, provides the ultimate resource for genomic approaches. Whole-genome resequencing data with positional information along a genome sequence constitute the most complete account of individual genomic variation [e.g. structural rearrangements, copy number variation, insertion–deletion, single nucleotide polymorphisms (SNPs), sequence repeats] and will likely soon become the standard for genetic studies of natural populations (Ellegren et al. 2012; The Heliconius Genome Consortium 2012). It also provides the basis for haplotype information and genomewide estimates of linkage disequilibrium which have great power to reveal recent population histories (Li and Durbin 2011), timing of admixture events (Hellenthal et al. 2014) and to screen for signatures of selection (Hohenlohe et al. 2010). The study of selectively important variation strongly relies on annotated genome data to identify the functional genomic regions of interest. Reference sequences are further indispensable as a template for RNA-seq in detailed studies of (isoform-specific, allele-specific) gene expression (Vijay et al. 2013), epigenetic modifications (such as methylation; Herrera and Bazaga 2011) and DNA–protein interactions (Auerbach et al. 2013). These approaches are only accessible to genome-enabled taxa (Kohn et al. 2006) that enjoy the added benefit of using the latest bioinformatics tools developed in the biomedical sciences.

Here, we introduce the workflow of a typical whole-genome sequencing project conducted by an individual research group. This field guide aims at introducing principles and concepts to beginners in the field (Box 2) and offers practical guidance for the many steps involved (Fig.1). It builds largely upon our own experience with vertebrate genome assembly. We limit the scope to genomic data, focusing on large and complex genomes, for transcriptome assembly we refer to Martin and Wang (2011) and Wolf (2013). We discuss sequencing, assembly and annotation, highlighting typical routines and analytical procedures. Our intention is not to provide a comprehensive review of sequencing technology, assembly algorithms or downstream downstream analyses, as this has already been performed. For these topics, we instead list exemplary literature and provide relevant entry points (Box 3).

**Box 1: Glossary**

**Table:** 

Alignment	Similarity-based arrangement of DNA, RNA or protein sequences. In this context, subject and query sequence should be orthologous and reflect evolutionary, not functional or structural relationships
Annotation	Computational process of attaching biologically relevant information to genome sequence data
Assembly	Computational reconstruction of a longer sequence from smaller sequence reads
Barcode	Short-sequence identifier for individual labelling (barcoding) of sequencing libraries
BAC	(Bacterial artificial chromosome) DNA construct of various length (150–350 kb)
cDNA	Complementary DNA synthesized from an mRNA template
Contig	A contiguous linear stretch of DNA or RNA consensus sequence. Constructed from a number of smaller, partially overlapping, sequence fragments (reads)
Coverage	Also known as ‘sequencing depth’. *Sequence coverage* refers to the average number of reads per locus and differs from *physical coverage*, a term often used in genome assembly referring to the cumulative length of reads or read pairs expressed as a multiple of genome size
*De novo* assembly	Refers to the reconstruction of contiguous sequences without making use of any reference sequence
EST library	Expressed sequence tag library. A short subsequence of cDNA transcript sequence
Fosmid	A vector for bacterial cloning of genomic DNA fragments that usually holds inserts of around 40 kb
GC content	The proportion of guanine and cytosine bases in a DNA/RNA sequence
Gene ontology (GO)	Structured, controlled vocabularies and classifications of gene function across species and research areas
InDel	Insertion/deletion polymorphism
Insert size	Length of randomly sheared fragments (from the genome or transcriptome) sequenced from both ends
K-mer	Short, unique element of DNA sequence of length k, used by many assembly algorithms
Library	Collection of DNA (or RNA) fragments modified in a way that is appropriate for downstream analyses, such as high-throughput sequencing in this case
Mapping	A term routinely used to describe alignment of short sequence reads to a longer reference sequence
Masking	Converting a DNA sequence [A,C,G,T] (usually repetitive or of low quality) to the uninformative character state N or to lower case characters [a,c,g,t] (*soft masking*)
Massively parallel (or next generation) sequencing	High-throughput sequencing nano-technology used to determine the base-pair sequence of DNA/RNA molecules at much larger quantities than previous end-termination (e.g. Sanger sequencing) based sequencing techniques
Mate-pair	Sequence information from two ends of a DNA fragment, usually several thousand base-pairs long
N50	A statistic of a set of contigs (or scaffolds). It is defined as the length for which the collection of all contigs of that length or longer contains at least half of the total of the lengths of the contigs
N90	Equivalent to the N50 statistic describing the length for which the collection of all contigs of that length or longer contains at least 90% of the total of the lengths of the contigs
Optical map	Genomewide, ordered, high-resolution restriction map derived from single, stained DNA molecules. It can be used to improve a genome assembly by matching it to the genomewide pattern of expected restriction sites, as inferred from the genome sequence
Paired-end sequencing	Sequence information from two ends of a short DNA fragment, usually a few hundred base pairs long
Read	Short base-pair sequence inferred from the DNA/RNA template by sequencing
RNA-Seq	High-throughput shotgun transcriptome (cDNA) sequencing. Usually not used synonymous to RNA-sequencing which implies direct sequencing of RNA molecules skipping the cDNA generation step
Scaffold	Two or more contigs joined together using read-pair information
Transcriptome	Set of all RNA molecules transcribed from a DNA template

**Box 2: Before you start**

**Table:** 

**Some important points to consider**	
• Availability of appropriate computational resources	
• Collaboration with sequencing facility and bioinformatics groups	
• Plan for amount and type of sequencing data needed	
• Does funding allow to produce sufficient sequence coverage? If not, alternative approaches should be considered rather than producing a poor, low coverage, assembly	
• Familiarization with data handling pipelines and file formats (see below)	
• High-quality DNA sample (with individual metadata)	
• Plan for analyses and publication	
**Some useful resources**	
*Internet forums for discussions related to genome sequencing*	
• http://seqanswers.com/	
• http://www.biostars.org/	
• http://www.biosupport.se/	
*Entry points to genome sequencing, assembly and exemplary downstream analyses*	
• Library preparation and Sequencing: Mardis (2008, 2013)	
• Quality filtering/preprocessing: Patel and Jain (2012), Zhou and Rokas (2014), Smeds and Künstner (2011)	
• Genome assembly: Nagarajan and Pop (2013), Pop (2009), Flicek and Birney (2009)	
• Assembly evaluation: Earl et al. (2011), Bradnam et al. (2013), Bao et al. (2011)	
• Genome annotation: Yandell and Ence (2012)	
• Mapping: Li and Durbin (2009), Trapnell and Salzberg (2009), Bao et al. (2011)	
• Data handling: Li et al. (2009), Quinlan and Hall (2010)	
• Variant calling: Nielsen et al. (2011), DePristo et al. (2011), Van der Auwera et al. (2013)	
• Haplotype-based approaches: Browning and Browning (2011), Tewhey et al. (2011), Lawson et al. (2012)	
• Population genomic summary statistics: Nielsen et al. (2012b), Danecek et al. (2011)	
*Web resources*	
• Galaxy (http://galaxyproject.org/)	
• Amazon cloud (http://aws.amazon.com/ec2/)	
• Windows Azure (http://www.windowsazure.com/)	
• Magellan: Cloud Computing for Science (http://www.alcf.anl.gov/magellan)	
• Web Apollo (http://genomearchitect.org/)	
• NCBI BioProject (http://www.ncbi.nlm.nih.gov/bioproject/)	
• Genomes OnLine Database (http://genomesonline.org/cgi-bin/GOLD/index.cgi)	
• ENSEMBL genome database (http://www.ensembl.org/index.html)	
• UCSC Genome Browser (http://genomebrowser.wustl.edu/)	
• fastQCtoolkit for data preprocessing (http://www.bioinformatics.babraham.ac.uk/projects/fastqc)	
*Genome size databases*	
• Plants: http://data.kew.org/cvalues/	
• Animals: http://www.genomesize.com/	
**Common file formats**	
• FASTA	Nucleotide sequence (file extension .fas or .fa)
• FASTQ	Nucleotide sequence including quality scores
• SAM	Sequence alignment
• BAM	Binary version of SAM
• GFF3	Annotation
• GTF	Annotation
• BED	Annotation
• VCF	Variant calling

## Basic considerations

Genome assembly is a challenging problem that requires time, resources and expertise. Before engaging in a genome sequencing project, it should thus be carefully considered whether a genome reference sequence is strictly necessary for the purpose in question. Genome sequences are merely a resource and in many cases will contribute very little *per se* to a problem in conservation biology. In case a genome draft is judged to be of significant value to address the problem at hand, it needs to be considered whether sufficient financial and computational resources are available to produce a genome of satisfactory quality. If funding is not available to obtain the appropriate read depth, it is advisable to utilize alternative approaches where possible (such as genotyping-by-sequencing or transcriptome sequencing), rather than settle for low-coverage whole-genome sequencing data. The latter would be a waste of funding, effort and time.

One important limitation of the current shotgun genome sequencing approaches that may be of particular importance in conservation biology is the fact that core genes with high conservation relevance, like immune genes of the MHC or olfactory receptor (OR) genes, are highly polymorphic and have many paralogs, which makes them particularly difficult to assemble. More generally, rapidly evolving genes or members of large gene families are often poorly represented in the final assembly and annotated gene set. Such regions and genes constitute a challenge even for very large sequencing projects of model organisms. If the project is not carefully planned from the start, there is a risk that the regions of highest interest to conservation biology will not be correctly represented in the final draft of the genome. Manual annotation and use of additional data, such as targeted sequencing of bacterial artificial chromosome (BAC) clones, will often be necessary to include such genomic regions in the assembly. If information on such preidentified candidate genes is the main aim of the study, it might even be more efficient to focus only on those regions rather than trying to sequence and assemble the whole-genome (see for example Wang et al. 2012).

### What does it mean to ‘sequence a genome’?

Ideally, a genome draft would represent the complete nucleotide base sequence for all chromosomes in the species of interest, a ‘physical map’ of its genetic content (as opposed to the ‘genetic or linkage map’ which establishes the order and recombination distances among genetic markers). However, in reality, there are a number of complications with the concept of a ‘genome sequence’. First, there is not one true sequence for a species because of individual genomic variation. In a single diploid individual, such variation will manifest itself in the form of heterozygous positions, insertion/deletion (InDel) polymorphism, copy number variation or small-scale rearrangements. Even cells from the same individual can differ in genomic content due to somatic mutations. The assembled genome sequence of an individual will also be only one representation of the total variation present in a species (paralleling the use of ‘type specimens’ for taxonomic classification). Generally, only a single individual is sequenced (Wheeler et al. 2008), but sometimes (like in the HUGO project) the genome represents a ‘consensus’ of a number of pooled samples (International Human Genome Sequencing Consortium 2004). Note, however, that in diploid and polyploid organisms, the genome assembly already reflects a consensus sequence of several chromosome sets and fails to capture haplotypic variation (for most current short-read based methods). Second, it is essentially impossible to sequence and assemble all nucleotides in the genome (Ellegren 2014). Large parts of DNA sequence, especially the heterochromatic regions around centromeres and telomeres and other highly repetitive regions, are not well-characterized even in mature genome assemblies like human or mice. Third, there will always be some degree of error in the characterized genome sequence, both on the level of individual nucleotides (stemming from sequencing errors) and in the ordering of sequence blocks (stemming from assembly errors). Forth, every genome assembly is the result of a series of assembly heuristics and should accordingly be treated as a working hypothesis.

### The principle of genome sequencing and assembly

**Figure 2:**
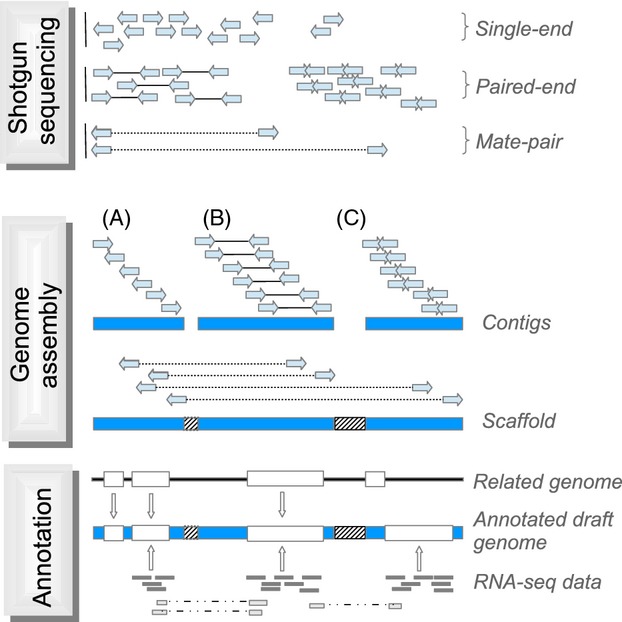
Simplified illustration of the assembly process and terminology. Shotgun sequencing: short fragments of DNA from the target organism are sequenced at random positions across the genome to a given depth of coverage. Fragments can consist of single reads (typically 50–1000 bp) or of paired-end reads of varying insert size (note that paired-end reads can even overlap). Mate-pair libraries span larger genomic regions (∼2–20 kb inserts) with reads generally facing outwards and can be complemented with fosmid-end libraries (∼40 kb inserts). Genome assembly: (A) short-read *de novo* assemblers extend the disperse sequence information from the reads into continuous stretches called *contigs*. *Contigs* usually reflect the consensus sequence and do not contain any polymorphisms. (B) Paired-end reads provide additional information on whether a read is supported for a given *contig*. (C) Some assemblers such as ALLPATHS-LG work with overlapping read pairs that are joined into a virtual longer read prior to the assembly. Read pairs from mate-pair or fosmid-end libraries can be used to order and orient *contigs* into *scaffolds*. Gap size between *contigs* is estimated from the expected length of mate-pairs and marked with ‘N's (indicated by hatched grey boxes). Long reads from single molecule sequencing provide an alternative. Annotation: gene models can be inferred *in silico* by prediction algorithms, by lifting over information from genomes of related organisms and by using transcriptome data (RNA-seq, expressed sequence tag) from the target organism itself. Spliced reads from RNA-seq data as indicated at the bottom of the figure provide valuable evidence for splice junctions and various isoforms of a gene.

Currently, most genome projects use a shotgun sequencing strategy for genome sequencing (Fig.2). In a first step, genomic DNA is sheared into small random fragments. Depending on the technology, these are sequenced independently to a given length. Powerful computer algorithms are then utilized to piece the resulting sequence reads back together into longer continuous stretches of sequence (*contigs*), a process known as *de novo* assembly. For correct assembly, it is important that there is sufficient overlap between the sequence reads at each position in the genome, which requires high sequencing coverage (or read depth). Naturally, for longer sequence reads, more overlap can be expected, reducing the required raw read depth. Usually, longer fragments (several hundred base pairs) are sequenced from both ends (paired-end sequencing) to provide additional information on correct read placement in the assembly.

After the initial assembly, *contigs* are typically joined to form longer stretches of sequence (known as *scaffolds*). To achieve this, libraries from long DNA fragments spanning several kilobases (kb) of sequence in the genome are prepared and their endpoints sequenced. Depending on the technology and the specifics of the library preparation, these libraries are (somewhat confusingly) called, for example paired-end, mate-pair or jump libraries. If the endpoint sequences of several independent fragments come to lie on two different *contigs*, they are joined into a *scaffold*. The expected fragment length of the library provides information on the physical distance between the two *contigs*, and the created gap is filled with the uninformative base-pair character ‘N’. Subsequent gap closing methods, ideally using long reads that read across repetitive sequences, help to fill in the missing base-pair information.

In a last step, the resulting *scaffolds* are often joined into linkage groups or placed on chromosomes (Ellegren 2014). Genetic maps constructed from pedigree data or crosses are arguably the best way for ordering and orienting *scaffolds* into longer sequence blocks (Ellegren et al. 2012). However, detailed genetic maps of species with conservation concern (usually not amendable to artificial crosses or half-sib breeding designs) require substantial genotyping effort, and deep pedigrees with a sufficient number of meioses are difficult to come by in most systems (Romanov et al. 2009). Given these difficulties, it is often not realistic to aim for a chromosome-level assembly, and this will also often not be necessary for most conservation biology applications. Most applications, including haplotype-based approaches that are powerful in revealing signatures of selection or depict recent demographic histories, generally work with high-quality *contigs*. As an alternative for placing and orienting the *scaffolds* onto putative chromosomes, synteny and gene order information from related species can be used. Note, however, that such information should be used with due caution as chromosomal rearrangements may have occurred even between very closely related species. There is also a risk that errors in the reference species assembly are transferred to the focal genome.

## Genome sequencing

### Sequencing technology and coverage considerations

Among the first decisions when starting, a genome sequencing project is the choice of sequencing platform, the type and amount of sequence data to generate. The latter is often limited by project funding, and the former may depend on which sequencing technology is promptly available. Judging from recently completed whole-genome sequencing projects (Table1), there is a clear trend moving away from traditional Sanger sequencing (∼1 kb sequence reads) and Roche 454 sequencing (up to 800 bp) towards short read technologies such as Illumina HiSeq (at present typically 150 bp) and SOLiD (typically 50 bp). Lately, there has been progress in producing longer reads at high throughput; several technologies offering this, such as Pacific Biosciences (up to 5 kb), IonTorrent (∼500 bp) and Illumina Moleculo (up to 10 kb), are entering the market, and we expect to see a broader spectrum of read lengths. While this development blurs the initial dichotomy of short reads (e.g. 35 bp Illumina reads) versus long reads (∼1 kb Sanger reads), read length still has important bioinformatic implications, as assembly algorithms optimized for long reads are fundamentally different from approaches targeting short reads. Recent studies begin to combine data of different read length and from several different sequencing platforms (Koren et al. 2012). This strategy makes intuitive sense as the drawbacks of each method can be counterbalanced, although the jury is still out whether such hybrid assemblies always outperform single data type approaches (Bradnam et al. 2013). Here, we follow the principle of current common practice and base our considerations largely on sequencing of Illumina libraries of different lengths (we loosely refer to short reads at sequence lengths below 500 bp and long reads above this length). Many of the following reflections, however, more generally relate to the assembly problem and do not depend on the specific choice of sequencing library.

**Table 1:** Some recently sequenced vertebrate genomes in species of conservation concern.

Species	Red list category	Sequencing technology	Assembly algorithm	Contig N50 (bp)	Sequencing coverage	Number of authors	References
Chimpanzee	EN	Sanger	PCAP	53000	6×	67	Consortium (2005)
Mammoth	EX	Roche 454	NA	NA	<1×	22	Miller et al. (2008)
Panda	EN	Illumina GA	SOAPdenovo	39886	56×	123	Li et al. (2010)
Orang-utan	CR	Sanger	PCAP	15654	6×	101	Locke et al. (2011)
Cod	VU	Roche 454	Newbler	2778	40×	42	Star et al. (2011)
Tasmanian devil	EN	Roche 454/Illumina GAIIx	Newbler/CABOG	9495	14×	30	Miller et al. (2011)
African elephant	VU	Sanger (ABI3730)	ARACHNE (reference assisted)	2900	2×	60	Lindblad-Toh et al. (2011)
Tarsier	NT	Sanger (ABI3730)	ARACHNE (reference assisted)	2900	2×	60	Lindblad-Toh et al. (2011)
Polar bear	VU	Illumina HiSeq 2000	SOAPdenovo	3596	100×	26	Miller et al. (2012)
Puerto Rican parrot	CR	Illumina HiSeq 2000	Ray	6983	27×	14	Oleksyk et al. (2012)
Gorilla	CR	Sanger/Illumina	Phusion assembler/ABySS	11800	50×	71	Scally et al. (2012)
Bonobo	EN	Roche 454	Celera Assembler	67000	25×	41	Prufer et al. (2012)
Yak	VU	Illumina HiSeq 2000	SOAPdenovo	20400	65×	48	Qiu et al. (2012)
Aye-aye	NT	Illumina GAIIx	CLC bio Assemler	3650	38×	10	Perry et al. (2012)
Coelacanth	CR	Illumina HiSeq 2000	ALLPATHS-LG	12700	61×	91	Amemiya et al. (2013)
Saker falcon	EN	Illumina HiSeq 2000	SOAPdenovo	31200	113×	25	Zhan et al. (2013)
Tibetan antelope	EN	Illumina GAIIx	SOAPdenovo (reference assisted)	NA	Not reported	11	Kim et al. (2013)
Bluefin tuna	LC*	Roche 454/Illumina GAIIx	Newbler/Bowtie	7588	54×	24	Nakamura et al. (2013)
Darwin's finch	LC†	Roche 454	Newbler	Not reported	4×	19	Rands et al. (2013)
Straw coloured fruit bat	NT	Illumina HiSeq 2000	CLC bio	27140	17×	7	Parker et al. (2013)
King cobra	VU	Illumina GAIIx	CLC/SSPACE	3980	40×	36	Vonk et al. (2013)
Burmese python	VU	Roche 454/Illumina HiSeq 2000	Newbler/SOAPdenovo	10700	49×	39	Castoe et al. (2013)
Chinese softshell turtle	VU	Illumina HiSeq 2000	SOAPdenovo	22000	106×	34	Wang et al. (2013)
Tiger	EN	Illumina HiSeq 2000	SOAPdenovo	29800	118×	58	Cho et al. (2013)
Minke whale	LC‡	Illumina HiSeq 2000	SOAPdenovo	22571	128×	55	Yim et al. (2014)
Northern bobwhite	NT	Illumina HiSeq 2000	CLC	45400	142×	12	Halley et al. (2014)
Black grouse	LC§	SOLiD 5500xl	SOAPdenovo (reference assisted)	1238	127×	5	Wang et al. (2014)
White rhinoceros	NT	Illumina HiSeq 2000	ALLPATHS-LG	93000	91×	10	Di Palma et al. unpublished data

Red list categories: EX, extinct; CR, critically endangered; EN, endangered; VU, vulnerable, NT, near threatened; LC, least concern.

* Not red-listed, but likely to be affected by overfishing.

† Not red-listed, but endemic to a small geographic region.

‡ Not currently red-listed but, subject to extensive exploitation or within group of endangered taxa.

§ Not globally red-listed, but with several small and isolated regional populations.

For most downstream applications, obtaining long *contigs* is essential. With long-read data, from traditional Sanger sequencing of individual BAC clones, this is feasible even with a rather limited sequencing depth. However, when using only short-read technologies, high total read coverage (>100×) is needed. Too little data will result in a highly fragmented assembly and severe problems with downstream applications such as annotation and variant calling. For initial *contig* assembly, one usually starts out with a high amount of paired-end short-read data. To subsequently merge *contigs* into *scaffolds*, it is necessary to generate additional libraries with long-insert sizes in the range of 3–40 kb (Fig.2). How much sequencing data are needed of each library types and insert size depends critically on a number of factors including the size and repeat content of the genome, the degree of heterozygosity and the target quality of the assembly (Sims et al. 2014). As these parameters will differ between sequencing projects and organisms of interest, the optimal resource allocation will be unique to every project. As a rough guideline for mammalian genomes, it has been proposed to use at least 45× coverage of short-insert paired-end libraries, 45× coverage of medium-sized insert libraries (3–10 kb) and 1–5× coverage of long-insert libraries (10–40 kb) (Nagarajan and Pop 2013). It should be noted that coverage can sometimes also be too high, as the absolute number of sequencing errors increases as a function of read number. According to our own experience, down-sampling from 100× to 50× coverage of a short-insert size library can significantly improve some steps in the assembly process.

To translate these recommendations into amount and type of sequencing needed for a specific project, basic knowledge on genome size, sequencing error rates, repeat content and the degree of genome duplications is needed. If no such information is available for the target species of interest at the start of the project, it is advisable to first perform a small pilot study using single-end or short-insert sequencing. The above-mentioned parameters can then be approximated using a k-mer counting approach (Marçais and Kingsford 2011; http://josephryan.github.com/estimate_genome_size.pl). Information on how to perform and interpret such k-mer counts can be found in web forums such as seqanswers (Box 2). Generally, a larger amount of long-insert data is needed for correct assembly if the genome has a high repeat content or a high degree of duplications. Genome size estimates for a large number of species are also available in online databases (Box 2).

### Wet-lab procedures

The wet-lab part of the genome sequencing is often outsourced to sequencing centres, and we will only very briefly touch upon the basic steps of library preparation that are important to consider at the planning stage of the project and that affect downstream analytical procedures.

#### Genome individual

Heterozygous positions in the genome of the sequenced individual have adverse effects on the assembly. Highly polyploid species are particularly challenging for assembly and may necessitate specifically tailored assembly pipelines (Schatz et al. 2012). A general recommendation is to use inbred individuals, parthenogenetic or gynogenetic offspring where available. Attached to the genome individual should be metadata that might be important for future referencing, such as the identity, age and sex of the individual, time and exact place of sampling, etc. (Genome 10K Community of Scientists 2009).

#### Tissue

Energetically active tissue (such as muscle) should be avoided, as there is a risk that the sequence data will contain a high proportion of mitochondrial DNA (mtDNA), which wastes sequencing effort and can cause problems in the assembly step due to the extreme read depth (as assembly pipelines often use read depth to identify duplicated genomic regions). We further recommend removing mtDNA sequence reads prior to assembly and use only a small fraction of this data to assemble the mitochondrial genome (which in itself may provide important information for conservation genetics) separately. It is also advisable to avoid tissues such as gut and skin which may have a high degree of nontarget DNA from xenobiotic organisms.

#### DNA quality

Whole-genome sequencing, particularly of long-insert size libraries, requires high-quality, intact, nondegraded DNA at a sufficient amount (Wong et al. 2012). For sequencing, a full genome using a set of different libraries requires ∼1 mg of DNA as starting material (∼6 μg for short-insert libraries, ∼40 μg for 2–10 kb libraries, ∼60 μg for >20 kb libraries). Before engaging in genome sequencing, it is thus essential to obtain a large amount of high-quality DNA of the target species. This can be a major obstacle for many species with conservation concern. If captive animals are available, such samples can often be utilized as a source of high-quality DNA, but note that genomic variation identified from such sources may not be representative of wild populations. Prior to submitting a DNA sample, its integrity should be checked on a high-resolution gel (e.g. pulse-field electrophoresis; a sample should typically show fragments of >100 kb).

#### Library preparation

When choosing the necessary raw read depth, one should be aware that currently most technologies include several PCR steps which can lead to a non negligible number of duplicated reads. While single reads can occur in duplicate by chance if coverage is high enough, duplication is bound to be an artefact for identical read pairs which are very unlikely to occur by chance (as they follow a length distribution). As duplicated reads are of no added value and duplication artefacts can impair coverage-based quality validation, they should be removed prior to the assembly. Duplicates generally constitute a few percentage of short-insert size libraries (<500 bp), but can reach over 95% for long-insert libraries (>10 kb).

Another central question refers to what insert sizes to use. Generally, it is advisable to have a good mix of sizes in the range of 0.2–40 kb with the shorter libraries being sequenced to significantly higher depth (Gnerre et al. 2011). Insert sizes of >20 kb make a large difference to the final contiguity and *scaffold* size of the assembly, but are not trivial to produce at high quality and currently constitute a limitation of many sequencing centres. Library preparations differ in quality and in how well they represent different parts of the genome. Therefore, more than one library should ideally be generated per size class. Note that some assembly programs (such as ALLPATHS-LG) expect a predefined mix of sequencing libraries as input data. Another important issue for downstream analyses that comes with library preparation is read orientation. Depending on the technology used, reads can face inwards (→ ←; e.g. Illumina paired-end sequencing) or outward (← →; e.g. Illumina mate-pair sequencing) in relation to the original DNA fragment. Mis-oriented reads with unexpectedly short insert sizes can arise due to sequencing of pairs from within the original DNA fragment rather than at its ends. Also, mate-pairs with aberrant insert sizes and orientation often represent chimeric sequences from nonadjacent genomic regions. For most assembly methods, such artefacts need to be filtered out during the preassembly steps, often leaving only a small fraction of usable, unique read pairs for assembly after trimming. To correctly process the data, the bioinformatician handling the data always needs to be ‘library aware’.

## Genome assembly

### Data management

The amounts of data generated in a normal genome sequencing project is staggering. A vertebrate genome with 100× coverage means data files in the order of several hundred gigabytes. During the assembly procedure, temporary files easily cross the terabyte boundary. An adequate data management and backup strategy is thus needed already at the start of the project. Many universities are connected to local or national computing grids, including data storage facilities, and it is highly recommended to utilize these whenever possible. Having bioinformatics application experts working at the computing infrastructure provides a vital link between the biologist researchers and the computing grid system experts (Lampa et al. 2013). Such collaborations should already be established during the planning stage of the project. Sequencing centres often also offer assistance with data analyses and assembly. However, their automated pipelines are not likely to be optimized for data from nonmodel organisms and might not be usable from a conservation biology point of view. It is thus vital to explicitly discuss what kind of support can be provided by the facility before the start of the project. More generally, it should be considered whether enough expertise exists in the core research group to perform the computational steps of an assembly. Most data processing and genomic analyses of large-scale sequencing data are conducted on high-performance computing clusters running a UNIX-based operating system. One does not need to be a bioinformatics expert to handle whole-genome sequencing data, but is essential to have some familiarity with the UNIX environment and basic knowledge in command line software, writing shell scripts and applying scripts of commonly used languages for biological data analysis (such as Perl or Python).

### Preassembly steps

Prior to the assembly, the quality of the sequencing data, overall GC content, repeat abundance or the proportion of duplicated reads should be assessed. Tools such as FastQC (http://www.bioinformatics.babraham.ac.uk/projects/fastqc) providing summary statistics are a useful starting point. Trimming low-quality data and reads resulting from PCR duplications can be performed with a variety of different software and scripts (e.g. ConDeTri; Smeds and Künstner 2011). Stand-alone error correcting, using a k-mer count approach (for example as applied in the SOAPdenovo pipeline), can also be a useful alternative for many datasets. Note, however, that the optimal stringency of quality filtering is specific to the individual project and the targeted assembly pipeline. Some assemblers, such as ALLPATHS-LG (Gnerre et al. 2011), where trimming and error correction are performed within the assembly pipeline, even require raw reads, without quality trimming as input.

Primer and vector sequences from the library preparation will most likely be present in the data (even if the sequencing facility claims to have removed them) and can be removed with simple scripts (like cutadapt; Martin 2011). Also, in Illumina sequencing, DNA from the PhiX phage is often added to the sequencing reaction, in order to calibrate sequence quality scores. Failure to remove such abundant contaminant sequences can disrupt the assembly process (due to the high read depth compared with the nuclear genome) and may result in the production of chimeric and contaminated *contigs*. The easiest way of removing known vector contamination from the raw data is to use a short read aligner (like BWA; Li and Durbin 2009) and delete all fragments mapping to the contamination sequence.

### *De novo* assembly

Tools for genome assembly differ widely in their performance in terms of speed, scalability and the quality of the final genome sequence (Miller et al. 2010; Earl et al. 2011; Narzisi and Mishra 2011; Bradnam et al. 2013). While some assembly methods clearly outperform others, it is currently difficult to predict which of the tools might be most appropriate in a given situation. Every assembly project is unique in terms of generated data structure and the target genome differing, for instance, in size, base-composition, repeat content and polymorphism level. There are a number of software available for *de novo* assembly of shotgun whole-genome sequencing data, and new programs are constantly being added to the list. Some algorithms focus on minimizing mis-assemblies, while others mainly aim to improve contiguity (sometimes at the cost of accuracy). Most assembly algorithms perform optimally with a given distribution of library sizes, so it is important to consider the choice of assembly strategy already during the project planning and sequencing steps. Besides information from the primary literature and websites of assembly software, various web forums provide good entry points for up-to-date discussions and sharing the experiences of other researchers (see Box 2).

Most software implementations designed for long-read technologies such as traditional Sanger sequencing (for example the Celera assembler, Arachne and PCAP; Batzoglou et al. 2002; Huang et al. 2003; Denisov et al. 2008) or Roche 454 sequencing (for example Newbler) use an assembly approach known as overlap-layout-consensus (OLC). These algorithms are generally considered too computationally intensive (mainly in terms of runtime) for Illumina or SOLiD data. Still, a few assemblers such as Edena (Hernandez et al. 2008), SGA (Simpson and Durbin 2012) and FERMI (Li 2012) pursue the OLC strategy for such short-read data (Miller et al. 2010). Most other strategies for *de novo* assembly of short sequence reads can be broadly divided into two classes: extension-based methods and De Bruijn (or Eulerian) graph algorithms (Nagarajan and Pop 2013). Extension-based assemblers, such as SSAKE (Warren et al. 2007) and JR-Assembler (Chu et al. 2013) are usually computationally very efficient (in terms of both memory requirements and computational time), but are highly sensitive to sequencing errors, repeat regions and high levels of nucleotide polymorphism (Chu et al. 2013). The most commonly used approach for assembly of short-read data is therefore currently based on De Bruijn graphs, where reads are partitioned into k-mers (substrings of the read sequence of length k) that then form the nodes of the graph (network) and are linked when sharing a k-1 mer. Highly used assembly software, such as SOAPdenovo (Luo et al. 2012), ALLPATHS-LG (Gnerre et al. 2011), ABySS (Simpson et al. 2009) and Velvet (Zerbino and Birney 2008), all rely on De Bruijn graph algorithms. There are also ‘hybrid’ assembly approaches, for example Atlas (Havlak et al. 2004), Ray (Boisvert et al. 2010) and MaSuRCA (Zimin et al. 2013), combining features of different algorithms and utilizing data from multiple sequencing technologies. In general, it is advisable to test several different assembly methods and evaluate which is most appropriate for the specific data at hand. Draft genome building should be treated as an iterative process with several rounds of assembly, evaluation and parameter tweaking. For a more comprehensive review of different assembly algorithms and software; see for example (Miller et al. 2010; Nagarajan and Pop 2013).

After the initial *contig* building, it is common to use read-pair information from long-insert (mate-pair, fosmid-end or jump) libraries (Zhang et al. 2012) to combine *contigs* into *scaffolds*. Additional short-insert paired-end libraries are also often useful, for example to bridge, short low-complexity regions. The lengths of sequence gaps between *contigs* are estimated from the expected insert sizes and are usually filled with a stretch of Ns. The scaffolding step is already included in many commonly used assembly programs, but there are also some stand-alone applications, for example SSPACE (Boetzer et al. 2011) and BESST (Nystedt et al. 2013), to perform this step independently. Some of the gaps (N's) emerging from this process can be removed a posteriori using the original read-pair information with software such as GapCloser (Li et al. 2010), GapFiller (Boetzer and Pirovano 2012) and iMAGE (Tsai et al. 2010). Long-read data (for example from PacBio) has also recently emerged as a way of filling N regions in *scaffolds* (English et al. 2012).

When choosing assembly software, it is important to consider both the amount of sequencing data and which computational resources are available (Schatz et al. 2010a). De Bruijn graph methods, such as SOAPdenovo and ALLPATHS-LG, generally require large amounts of computing memory (RAM). Depending on the amount of sequencing data, assembly of mammal-sized genomes (∼3 Gb) can require terabytes of internal memory (Lampa et al. 2013). If large computer clusters are not available locally, it will be necessary to consider collaborative equipment purchases, joint projects with bioinformatics groups or utilization of commercially available computing clouds (Box 2; Schatz et al. 2010b).

Another consideration to make is whether to use freely available programs (most programs mentioned above fall in this category) or to invest in commercial software (such as CLC workbench or Lasergene from DNASTAR). Commercial software is usually more user-friendly than freely available programs and thus readily used by researchers with limited bioinformatics skills. The downside of commercial software, apart from the (often substantial) cost involved in purchase and licensing, is that these act even more like ‘black box’ solutions, where it is often near impossible to inspect or alter details about the algorithms. Some commonly used software applications are also distributed together with the sequencing instruments and may be available through the sequencing facilities.

For an increasing number of species with conservation concern, there are genomic information available for very closely related taxa (Kohn et al. 2006). In such cases, it is an attractive alternative to use as much information as possible from the related genome in the assembly process (Gnerre et al. 2009). Such a ‘reference-assisted’ assembly strategy has been utilized in several studies (Table1), mainly using custom pipelines, and there is clearly great scope for development of more mature software in this field. The most common approach is to first produce *contigs de novo* and then align these to the genomic reference to aid in the scaffolding step. Assuming extensive synteny and gene order conservation, such an approach makes it possible to build large *scaffolds* even with low coverage data (Kim et al. 2013) or using very short sequence reads (Wang et al. 2014). An alternative approach is the so-called ‘Align-Layout-Consensus’ algorithm. Here, the overlap stage of the *de novo* assembly is replaced by alignment of reads to a closely related reference genome (which is computationally less intensive compared with the OLC approach). *Contigs* and *scaffolds* are then built *de novo*, using information from overlapping reads (Schneeberger et al. 2011).

### Quality assessment and validation

Once an assembly has been successfully performed, users will want to assess its quality or compare several assemblies using different methods. Yet, as discussed above, every draft genome assembly constitutes merely a hypothesis of the true underlying genome sequence, and in the absence of knowing the truth, assessing its quality remains a challenge.

A variety of metrics reflecting different aspects of the assembly are available (Bradnam et al. 2013). They can be broadly divided into approaches that require additional information from external data and those solely based on information derived from the assembly itself. As external information is often not available in conservation genomics projects, intrinsic quality assessment of the assembly is a natural starting point. One basic metric is the proportion of the genome contained within the assembly. The expected genome size can either be inferred from *C*-value data (Box 2) or, alternatively, from k-mer frequency-based approaches. Another standard metric to evaluate assembly contiguity is the N50 statistic: by definition, 50% of the assembled nucleotides are found in *contigs* (contig N50) or *scaffolds* (scaffold N50) of at least this length. The N50 statistic thus describes a kind of median of assembled sequence lengths, giving greater weight to long sequences. Recently, variations of this metric, for example the NG50 and ‘NG Graph’ (Bradnam et al. 2013), incorporating the expected genome size was introduced and provide effective means of visualizing and evaluating differences in contiguity between assemblies.

However, the N50 statistic and variations thereof need to be interpreted with caution. They merely indicate contiguity and contain no information on assembly accuracy. To detect errors in the assembly, information from remapped paired-end or mate-pair data can be used (as, e.g., implemented in the software REAPR; Hunt et al. 2013). Low-coverage regions or mis-orientation of read pairs suggests mis-assemblies, while aberrant insert sizes indicate small insertions or deletions. Exceedingly high sequence coverage, high local SNP densities or correlated SNPs, where most of the reads carry one character state (but multiple others show another character state), can indicate the presence of collapsed, near-identical repeats. Software applications performing these steps are numerous, and examples can be found in the current literature (Earl et al. 2011; Bradnam et al. 2013). The *amosvalidate* pipeline (Phillippy et al. 2008; Schatz et al. 2013) encompasses several genome assembly diagnostics in one pipeline, but works best for small- or medium-sized genomes.

Independent experimental data sets from the target species arguably provide the best source of external information. Data from optical maps, for instance, allow validating short- and large- scale accuracy of *scaffolds* and expanding them further to approach chromosome level. Similarly, separately assembled sequences from BAC or fosmid libraries can help to assess sequence accuracy and repeat content. Both approaches, however, rely on correct assembly themselves and are not readily available for smaller laboratories at present. Independent *de novo* assemblies from shotgun transcriptome sequencing data (RNA-seq) are more easily generated, and expressed sequence tag (EST) libraries might already exist for species of conservation concern (although getting access to fresh tissues for RNA extraction may be a serious limitation if captive populations are not available). Sequence content and exon structure of transcriptome data thus constitute an important additional resource for validating sequence accuracy and for correcting scaffolding in cases where genes span across *contigs*.

Comparative genomic approaches provide another avenue, which does not require the generation of additional data. For example, quantifying the presence and completeness of orthologous core eukaryotic protein sequences (Parra et al. 2007) provides first intuition on the comprehensiveness of the assembly. In cases where high-quality reference genomes of sister taxa exist, genome comparisons might be of guidance in detecting mis-assemblies and chimeric *contigs* under the assumption of broad-scale synteny and gene order conservation. Small-scale rearrangements, however, might be real and require in depth investigation.

DNA from other organisms are likely to have contaminated the genomic samples at various stages (during both sampling and laboratory procedures) and will be present in the sequencing data. Although mainly being a nuisance, contaminations at the sampling stage may actually be interesting from a conservation point of view, as they can carry information about parasites or other microorganisms related to the study species. External genomic resources aid in finding such contaminations that might have been assembled as separate *contigs* or are interspersed with target sequence in the same *contig*. Positive hits from a BLAST search or similar local alignment routines are often employed to find such traces of contamination, but results need to be interpreted with caution. Even correctly assembled sequences can lead to best hits from distantly related species with well-annotated genomes, particularly if taxon sampling within the target group of organisms is scarce. Likewise, small stretches of contamination in a large *contig* or *scaffold* may be missed entirely if other parts of the sequence yield significant hits on the target clade. Human contamination in other mammalian genome sequences will be particularly problematic, as such contamination is expected to be common due to handling of the samples. For parts of a *de novo*-sequenced mammalian genome, the best BLAST hit will be against a human or mouse sequence simply because the region in question has not been sequenced and annotated in any other mammal.

## Genome annotation

To harness the full potential of a genome sequence, it needs to be annotated with biologically relevant information that can range from gene models and functional information, such as gene ontology (GO) terms (Gene Ontology Consortium 2004; Primmer et al. 2013) or ‘Kyoto encyclopedia of genes and genomes’ (KEGG) pathways (Kanehisa and Goto 2000), to microRNA and epigenetic modifications (The ENCODE Project Consortium 2012). In the context of genetic nonmodel organisms, annotation is often confined to protein-coding sequence (CDS) or transcripts more generally. Despite the considerable challenge to annotate genes in newly sequenced species where preexisting gene models are mostly lacking, automated gene annotation has in principle become possible for individual research groups (Yandell and Ence 2012). Still, a complete genome annotation constitutes a considerable effort and requires bioinformatic proficiency. We describe only the general workflow and refer the interested reader to a comprehensive review by Yandell and Ence (2012) for more details (Box 2). Before starting, it should be noted that successful annotation strongly depends on the quality of the genome assembly. Only contiguous near-complete (∼90%) genomes interrupted only by small gaps will yield satisfying results. As a rule of thumb, large genomes have longer genes and thus need more contiguous assemblies for successful annotation (cf. Figure 1 in Yandell and Ence 2012).

The annotation process can be conceptually divided into two phases: a ‘computational phase’ where several lines of evidence from other genomes or from species-specific transcriptome data are used in parallel to create initial gene and transcript predictions. In a second ‘annotation phase’, all (sometimes contradicting) information is then synthesized into a gene annotation, following a set of rules determined by the annotation pipeline.

Prior to gene prediction, it is of vital importance to mask repetitive sequences including low-complexity regions and transposable elements. As repeats are often poorly conserved across species, it is advisable to create a species-specific repeat library using tools like RepeatModeler or RepeatExplorer (Novák et al. 2013). Once repeats are masked (e.g. with RepeatMasker; http://www.repeatmasker.org), *ab initio* algorithms trained on gene models from related species can be used for baseline prediction of coding sequence (CDS) (e.g. AUGUSTUS; Stanke et al. 2006). Protein alignments (using e.g. tblastx) and syntenic protein lift-overs from a variety of other species provide a valuable resource to complement the predicted gene models. Arguably, the best evidence comes from detailed EST or RNA-seq data, which in addition to CDS, provides gene models with information on splice sites, transcription start sites and untranslated regions (UTRs). If possible, mRNA should be sequenced strand-specifically, as this helps resolve gene models, facilitates transcriptome assembly and eventually aids in the evaluation of the genome assembly.

In a next step, all the evidence from *ab initio* prediction and protein-, EST- or RNA-alignments need to be synthesized into a final set of gene annotations. As the evidence is mostly incomplete and sometimes contradicting, this is a difficult task that often benefits from manual curation. Still, several automated annotation tools like MAKER (Cantarel et al. 2008) or PASA (Haas et al. 2003) exist that incorporate, and weigh the evidence from, several sources. Although these tools generally provide good results, qualitative validation is important (e.g. by assessing the length of open-reading frames). Visual inspection of the annotation is another vital component to detect systematic issues such as intron leakage (introns being annotated as exons due to the presence of pre-mRNA) or gene fusion. Tools like WebApollo (Lee et al. 2013) from the GMOD project are particularly useful, as they allow the user to edit the annotation directly through the visual interface.

### Publishing the genome

Draft genome sequences are now being produced at an ever-increasing rate. Traditional databases such as ENSEMBL from the European Molecular Biology Labs (EMBL) and the Wellcome Trust Sanger Institute, or genomic databases from the National Center for Biotechnology Information (NCBI) providing access to genomes and meta-information can no longer annotate and curate all incoming genomes. NCBI therefore already provides the possibility to upload draft genome sequences and user-generated annotation. To allow other users to improve the assembly and its annotation, all available raw data should be uploaded, together with the assembled genome and all relevant meta-data, for example as a BioProject on NCBI.

## Perspectives

### Conservation applications

We have summarized information on current methods for whole-genome sequencing, assembly and annotation, with the aim of providing practical guidance for conservation or ecology-oriented research groups moving into the field of genomics. The focus has been on large and complex genomes of nonmodel organisms relevant from a conservation perspective. In the introduction, we outlined a number different ways in which genomic resources in general, and a complete genome sequence, in particular, can be applied in a conservation biology setting (see also Fig.1). Conservation genomics being a young field, examples where genomic resources have been put to the test in an applied conservation context are still limited, but a few such cases may be worth highlighting.

One of the first nonmodel genomes to be sequenced using the Illumina technology was the giant panda (Li et al. 2010). While the focus of the panda genome paper was not on conservation issues, follow-up studies have utilized the draft genome to make inferences about population structure, adaptive genetic variation and demography (Wei et al. 2012). Likewise in the Aye-Aye, resequencing data from twelve individuals from different parts of Madagascar were utilized to infer fine-scale genetic population structure and conduct landscape genetic analyses. The results were used to provide guidance for allocation of conservation resources towards preserving large and contiguous habitats in northern Madagascar (Perry et al. 2013). Genomic resources have further been utilized in breeding programs of the Tasmanian devil, which is endangered in the wild due to a contagious facial cancer. The generation of a reference genome sequence in combination with genomewide resequencing data has made it possible to investigate many details of this disease, including the identification of candidate genes involved in tumorigenesis (Murchison et al. 2012). Similarly, genomic resources have been utilized to limit the spread of a developmental disease causing mutation in breeding programs of the California condor (Romanov et al. 2009). Finally, genomewide SNP screening has been effective in several studies of fishery stock monitoring and management (Primmer 2009; Nielsen et al. 2012a).

### Future directions

With rapid progress in sequencing nano-technology and further development of computational methods, we can expect that all steps of the workflow will continue to be improved. New library preparation protocols will enable sequencing from less starting material, producing libraries with longer and more precisely estimated insert sizes and generating longer reads with reduced error rates. The development of more efficient assembly algorithms and increasing computational power will make the bioinformatic data processing amenable to a larger spectrum of users. As the costs involved in genome sequencing and assembly continues to drop, the generation of a draft genome sequence will soon become routine, also for species with large genomes. This development will mean that even small research groups with limited funding will soon be expected to develop genomic resources for their species of choice, reinforcing the use of genomic approaches in conservation biology and related disciplines. The possible development of rapid and compact sequencing solutions that may be applied directly in the field situation would be particularly useful for many conservation applications. Another important area of progress lies in the usage of low-quality samples, obtained from noninvasive sampling or museum material that would allow monitoring of genomic diversity through time. Developing ways of storing and sharing genomic data will also be crucial, to make the most efficient use of these resources for conservation. In spite of these promising developments, we need to be aware that science alone is not sufficient to meet future conservation challenges. The technical transition from conservation genetics to genome-scale data therefore needs to be tightly accompanied by a discussion of how applied conservation biology can best benefit from genomic data (see for example McMahon et al., 2014). This discussion needs to be taken at the general level on a case-by-case basis and involves scientists and political decision makers alike.
